# Intrafamilial phenotypic variability in hypophosphatasia: evidence from two families and the literature

**DOI:** 10.3389/fendo.2026.1826307

**Published:** 2026-05-08

**Authors:** Elisa Sala, Ruggero Lanzafame, Katia Maruca, Stefano Mora, Marco Pitea

**Affiliations:** 1 Department of Pediatrics, IRCCS Ospedale San Raffaele, Milan, Italy; 2 University Vita-Salute San Raffaele, Milan, Italy; 3 Laboratory of Pediatric Endocrinology, Department of Pediatrics, IRCCS Ospedale San Raffaele, Milan, Italy

**Keywords:** familial hypophosphatasia, hypophosphatasia, phenotypic heterogeneity, tissue-nonspecific alkaline phosphatase, vitamin B6

## Abstract

**Background:**

Hypophosphatasia (HPP) is a genetic disorder caused by pathogenic mutations in the *ALPL* gene, resulting in reduced activity of the tissue-nonspecific alkaline phosphatase enzyme. The disease exhibits a wide phenotypic spectrum, varying from extremely severe forms that are often fatal during the prenatal or perinatal periods to very mild cases that are diagnosed in adult patients. Recent data indicate that familial cases account for approximately 50% of the total.

**Case presentation:**

We present two families showcasing significant phenotypic heterogeneity among siblings who share the same genotype. The first family proband exhibited skeletal deformities, bone hypomineralization, early tooth loss, and failure to thrive. In contrast, her older sister displayed only radiographic signs of hypophosphatasia (HPP). The medical history of the second family included a therapeutic abortion due to significant fetal skeletal malformations. The fetus was found to be compound heterozygous for pathogenic variants of the ALPL gene. A villocentesis conducted during a subsequent pregnancy revealed that the fetus had the same genotype. However, ultrasound examinations indicated that the fetus was developing normally and growing as expected. At birth, no overt skeletal malformations were observed; however, radiographs demonstrated hypomineralization, and laboratory findings were consistent with hypophosphatasia. The patient was subsequently diagnosed with craniosynostosis.

**Conclusions:**

Our case history emphasizes that, despite considerable progress in genetic diagnosis, predicting the severity of the phenotype remains difficult. Given the extreme phenotypic heterogeneity, even among family members, it is crucial to broaden the evaluation beyond prenatal findings by incorporating genetic investigations along with anamnestic, biochemical, and instrumental data.

## 1 Introduction

Hypophosphatasia (HPP) is a rare inherited disorder caused by loss-of-function variants in the *ALPL* gene, which encodes the enzyme tissue-nonspecific alkaline phosphatase (TNSALP). The prevalence of the disease is largely unknown due to difficulties in diagnosing patients with milder phenotypes (1). Genetic defects associated with the disease lead to reduced or absent enzyme activity in target tissues, resulting in the accumulation of TNSALP substrates such as inorganic pyrophosphate (PPi), pyridoxal-5’-phosphate (PLP, the plasma form of vitamin B6), and phosphoethanolamine (PEA). This accumulation impairs bone and tooth mineralization and, in severe cases, can cause vitamin B6-responsive seizures. The amount of residual enzymatic activity determines the wide spectrum of clinical manifestations, ranging from intrauterine or neonatal death to mild, non-specific musculoskeletal pain (1, 2). Although genotype–phenotype correlations in hypophosphatasia have been extensively investigated, the relationship between specific *ALPL* variants and disease severity remains only partially understood. Early studies (3, 4) suggested that mutations affecting key functional domains of the enzyme—such as the active site, homodimer interface, or metal-binding regions—are generally associated with more severe phenotypes, particularly in recessive forms. However, more recent evidence (5, 6) has highlighted substantial variability even among individuals carrying identical variants, indicating that genotype alone is often insufficient to predict clinical outcomes. For example, missense variants previously associated with severe disease have also been reported in patients with milder or late-onset forms, suggesting incomplete penetrance and variable expressivity. In addition, the presence of compound heterozygosity further complicates interpretation, as the functional interaction between variants may not be additive and can result in unpredictable enzymatic activity.

Although the disease spectrum is continuous, the classification was based on the age at which the first clinical manifestations appeared, regardless of the mode of inheritance of the pathogenic variants (7, 8). Seven clinical forms have been described: perinatal lethal, perinatal benign, infantile (severe and mild), childhood, adult, and odonto-HPP. A new classification based on genetic characteristics has been recently proposed (9). It consists of three subtypes: severe (corresponding to the perinatal and most infantile severe forms), moderate (infantile moderate, childhood, odonto-HPP, and adult with specific signs), and mild (adult with non-specific signs) (9). Inheritance of the severe subset is typically autosomal recessive, while inheritance of the mild form is mostly autosomal dominant. The moderate subset exhibits both recessive and dominant forms of inheritance. The latest classification, published in 2025, categorizes HPP into early-onset and late-onset types (10). This new method categorizes patients based on the age at which they first show symptoms of the disease. Early-onset patients are those who present before 6 months of age (with or without respiratory failure), while late-onset patients are defined as those who present at 6 months of age or older.

According to the Global HPP Registry, familial cases account for 43% of total cases (11). Despite sharing the same genotype, some family members exhibit different forms of the disease (12–14). The magnitude of this phenomenon remains unknown because the descriptions are very scarce. In this paper, we describe phenotypic heterogeneity in two familial cases of HPP. In our clinical practice, we have observed considerable phenotypic variability and the absence of a clear genotype-phenotype correlation. These observations prompted us to report this case series in order to contribute to a better understanding of this condition.

## 2 Case presentation

We present two families showcasing significant phenotypic heterogeneity among siblings who share the same genotype.

### 2.1 Family 1

The index case, a girl, presented with normal vital signs and growth parameters at birth, except for an unusual head shape. Brain ultrasound and neuromotor development were normal.

At the age of two, her pediatrician, during a routine screening, observed stunted growth, an unusual head shape, and a chest deformity. Consequently, he referred the girl to a medical geneticist to rule out any genetic syndrome. After performing radiological and biochemical exams, the geneticist referred the girl to our center.

**Figure 1:**
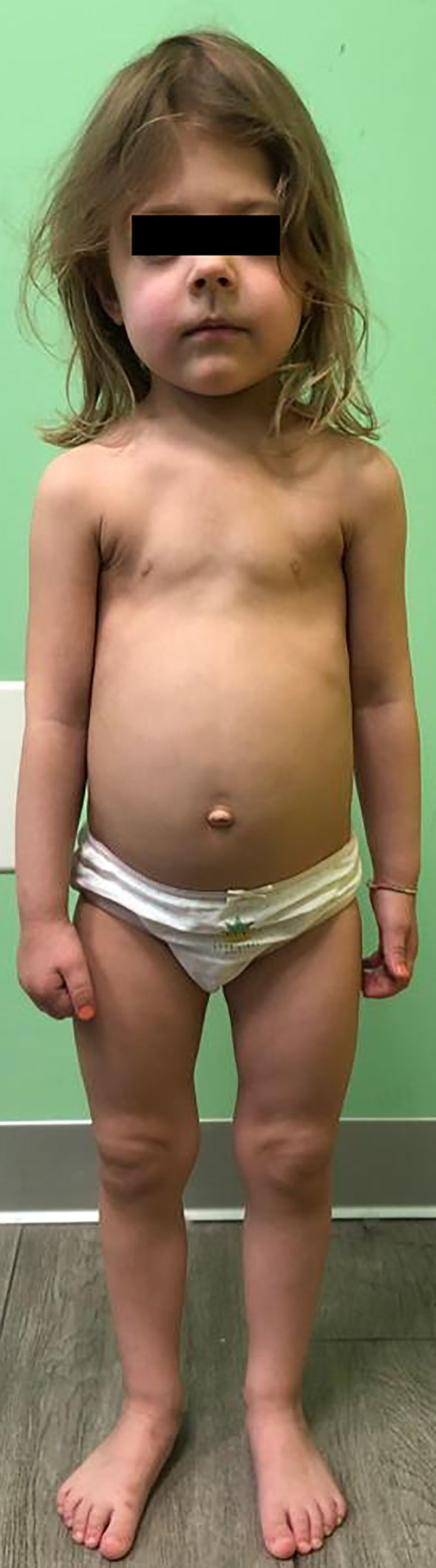
Clinical appearance of the proband from Family 1, a 3-year-old girl, at diagnosis of HPP. The girl showed craniomegaly and turricephaly, a bell-shaped thorax, and an axial deviation of the lower limbs. Premature loss of deciduous teeth with intact roots was observed.

The girl arrived at our observation at three years of age. Physical examination revealed the absence of upper and lower incisors, with intact roots, craniomegaly with turricephaly, a bell-shaped thorax, and axial deviation of the lower limbs (**Figure 1**). Her height was 87.5 cm (5th centile, -1.69 SDS according to WHO charts), her weight was 11.7 kg (5th centile, -1.61 SDS according to WHO charts), and her BMI was 14.29 kg/m^2^ (20th centile, -0.85 SDS according to WHO charts). According to the parents, the girl was not very active and frequently complained of fatigue. She showed a waddling gait.

Laboratory findings are shown in **Table 1**. We found a markedly low serum alkaline phosphatase activity (31 UI/L; normal reference for age and sex > 140 UI/L). Serum calcium and 25-(OH) vitamin D concentrations were within normal limits, while serum phosphate was slightly elevated compared to age-specific normal values. Parathyroid hormone concentrations were normal. Further laboratory tests showed very elevated serum vitamin B6 (282 ng/mL; normal range 12.6-45.2 ng/mL).

**Table 1:** Laboratory findings of Family 1 at diagnosis of HPP.

Variable	Proband	Sister	Father	Mother
Calcium (mg/dL)	9.85 (8.8-10.8)	9.74 (8.8-10.8)	9.24 (8.4-10.4)	9.35 (8.4-10.4)
Phosphate (mg/dL)	6.42 (3.8-6.5)	4.59 (2.9-5.4)	2.95 (2.5-4.6)	2.58 (2.5-4.6)
ALP (IU/L)	31^a^ (100-347)	28^a^ (100-315)	24^a^ (43-128)	74 (33-98)
PTH (pg/mL)	26.8 (15-65)	32 (15-65)	41.2 (15-65)	39.4 (15-65)
25OHD (ng/mL)	24.5 (20-60)	23.1 (20-60)	20 (20-60)	32.3 (20-60)
Vitamin B6 (ng/mL)	281.7^b^ (8.5-27.0)	258. ^b^ (8.5-27.0)	83.8^b^ (8.5-27.0)	31.9^b^ (8.5-27.0)

ALP (alkaline phosphatase), PTH (Parathyroid hormone), 25OHD (25-hydroxy-vitamin D). Reference ranges are in parentheses below the actual value. ^a^Markedly lower than the reference for age and sex. ^b^Markedly higher than reference.

**Figure 2:**
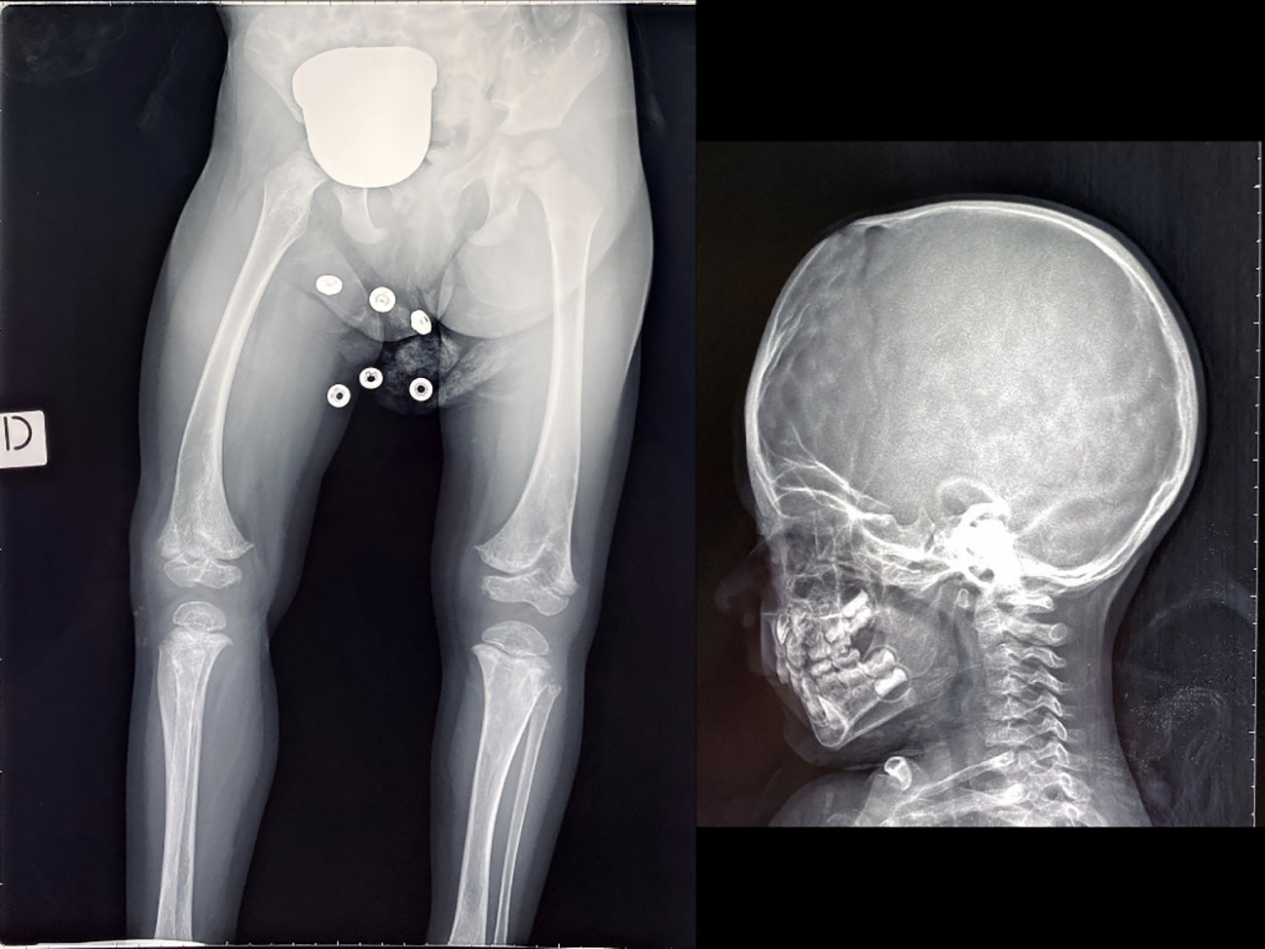
The radiographs of the proband from Family 1 showed subchondral areas of radiolucency in the distal femoral metaphysis, accompanied by irregularities in the metaphysis and generalized bone demineralization. The cranial vault exhibited marked mineralization defects with a distinctive "beaten copper" appearance.

Radiographic findings included subchondral tongues of radiolucency in the distal femoral metaphysis, metaphyseal irregularities, and generalized demineralization. The cranial vault demonstrated severe mineralization defects with a characteristic “beaten copper” appearance. Representative radiographs are presented in **Figure 2**.

The cranial deformation was studied by MRI, which showed a preserved brain morphology and confirmed turricephaly with differences in the thickness of the cranial teca.

Given the patient’s metabolic and radiographic abnormalities, genetic testing was performed. Sanger sequencing of the *ALPL* gene (NM_000478.6) revealed that the patient was a compound heterozygote for two variants. She was carrying the c.571G>A variant, causing amino acid substitution p.(Glu191Lys), and c.1258G>A, causing amino acid substitution p.(Gly420Ser), both classified as pathogenetic for HPP (**Table 2**) (15).

**Table 2:** Variants classification.

Family	Variant	Protein change	Classification	ACMG/AMP criteria
Family 1	c.571G>A	p.(Glu191Lys)	Pathogenic -Class 5	PS3, PM1, PM2, PP3, PP4
	c.1258 G>A	p.(Gly420Ser)	Pathogenic -Class 5	PS3, PM1, PM2, PM5, PP3
Family 2	c.407G>A	p.(Arg136His)	Pathogenic -Class 5	PS1, PS3, PM2, PP3
	c.1489T>C	p.(Cys497Arg)	Likely Pathogenic - Class 4	PM1, PM2, PM5, PP3

PS1, Same amino acid change previously established as pathogenic; PS3, Functional studies show severely reduced enzymatic activity; PM1, the variant is located in a highly conserved region of the protein, which is essential for its function or structural stability PM2, Absent/rare in healthy control populations; PM5, Alternative pathogenic variant described at the same codon PP3, Multiple in silico tools predict a deleterious effect.

The investigation was extended to the other family members. The mother reported musculoskeletal pain, fatigue, and stiffness, lasting several years. For this reason, she was undergoing a rheumatological follow-up, and she was on immunosuppressive treatment. The father was apparently healthy, with an unremarkable medical history. Both parents had a height above the 50th centile for the Italian population. The older sister was growing appropriately and did not present dysmorphic features.

All family members underwent measurements of alkaline phosphatase activity and vitamin B6 concentration. While the mother had measurements within the reference range, both the sister and father had low alkaline phosphatase activity and high vitamin B6 concentration. Laboratory findings of the whole family are reported in **Table 1**.

**Figure 3:**
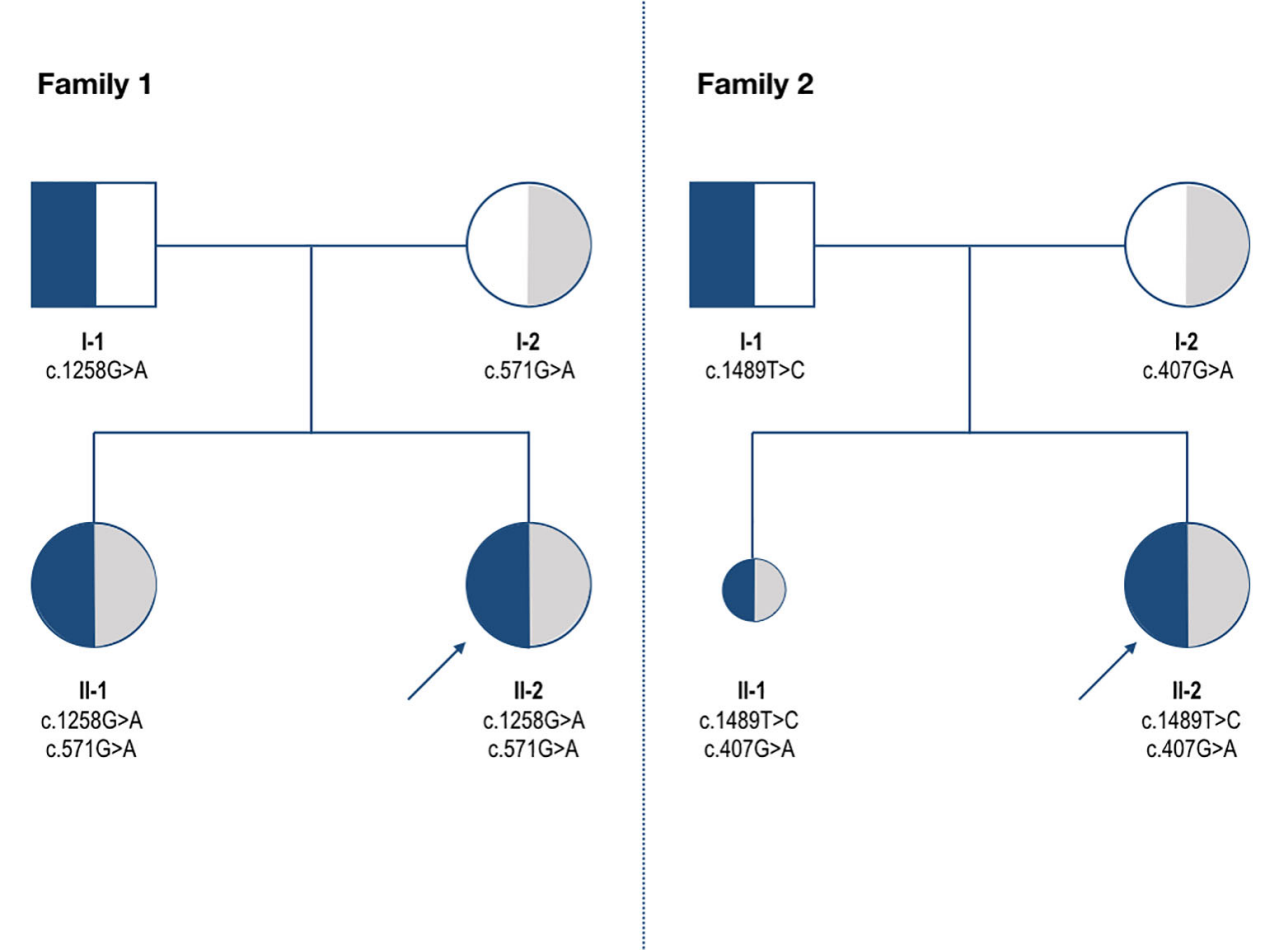
Pedigrees of Family 1 and Family 2. Genotypes (*ALPL* variants) are reported below each individual. In Family 1, both siblings are compound heterozygous for the c.571G>A and c.1258G>A variants, inherited from the mother and father, respectively. In Family 2, both parents are heterozygous carriers of distinct variants (c.407G>A and c.1489T>C). The offspring carry the same compound heterozygous genotype but exhibit discordant phenotypes, with a severe prenatal presentation in the first pregnancy and a milder postnatal phenotype in the second. The arrow indicates the proband.

Genetic testing was extended to the family members, and the family pedigree is shown in **Figure 3**. The older sister had the same genotype as the proband. The c.571G>A variant was inherited from the mother, while the father was the carrier of the c.1258G>A variant.

**Figure 4:**
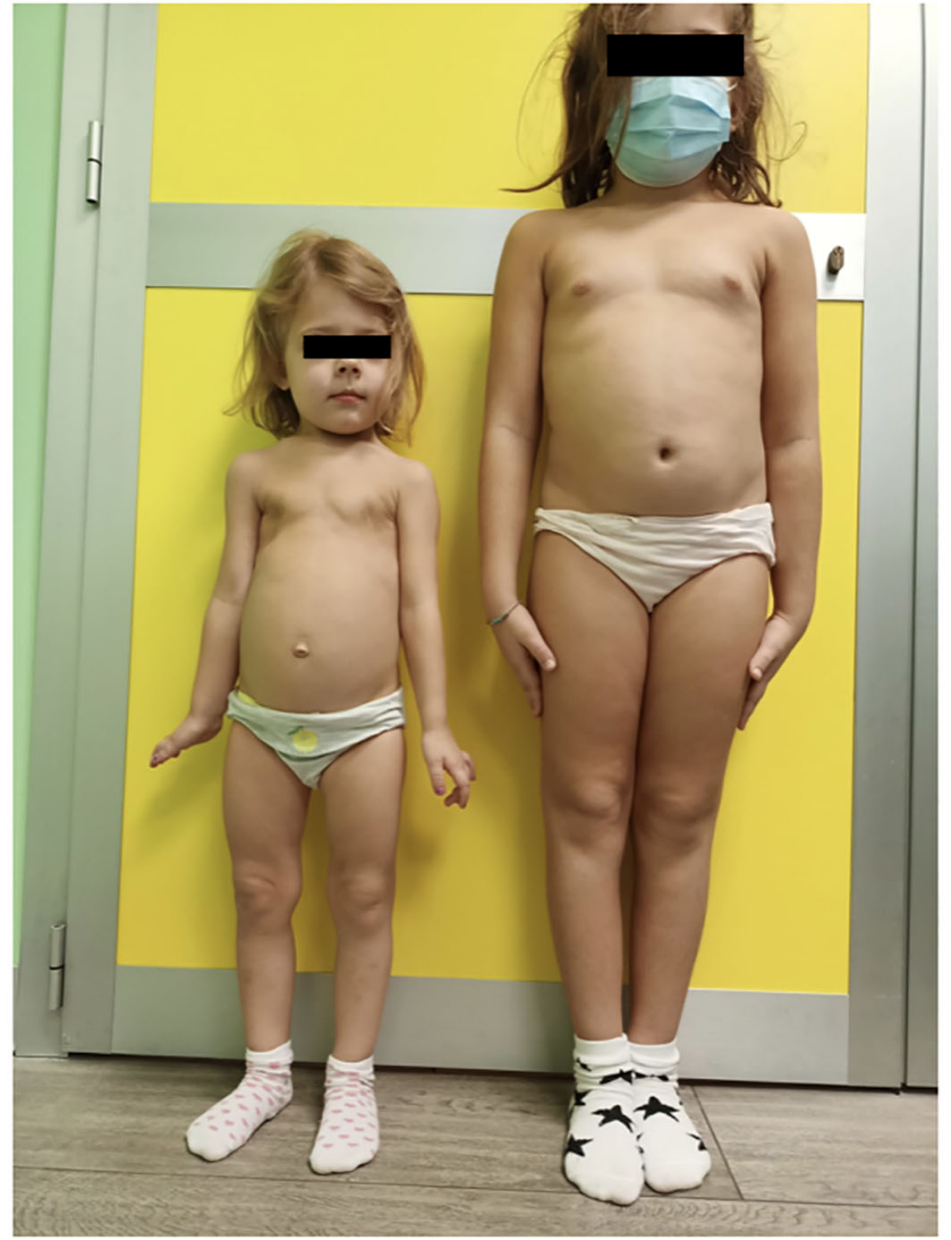
Phenotypic variability between affected siblings (Family 1). The proband shows overt clinical manifestations, while her sister exhibits a milder phenotype despite sharing the same genotype.

**Figure 5:**
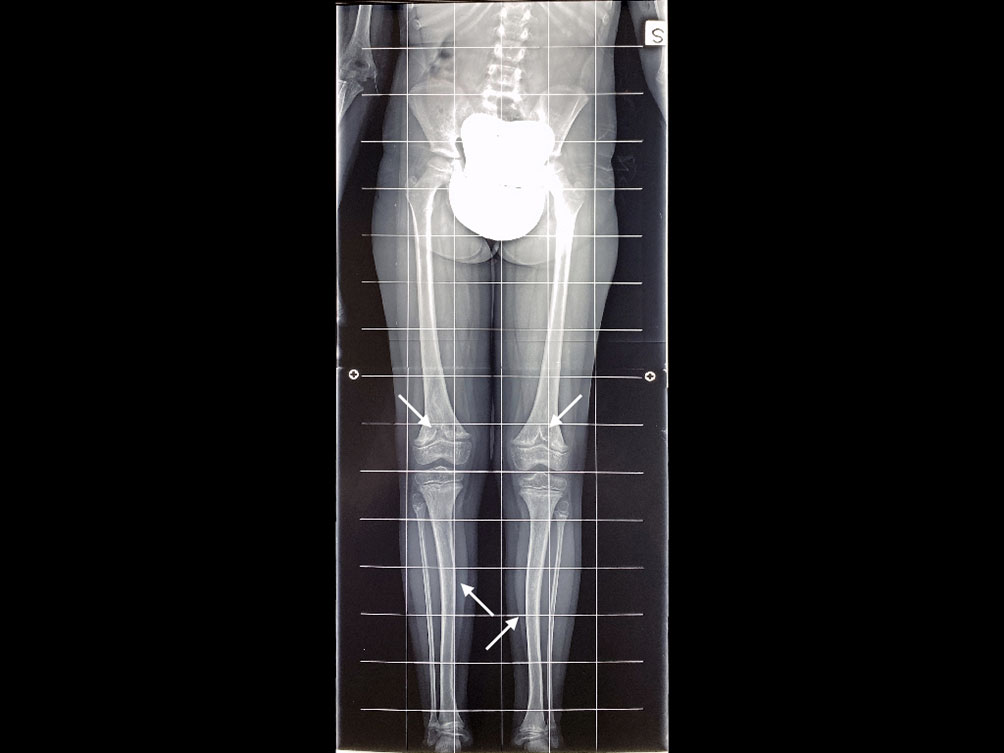
Radiographic findings in the elder sister (Family 1). Imaging reveals tibial deformities and metaphyseal irregularities, consistent with hypomineralization, despite the absence of overt clinical symptoms.

At diagnosis, the older sister was 7 years old and exhibited normal growth and development without suggestive symptoms (**Figure 4**). Her height was 116.2 cm (17th centile, -0.97 SDS according to WHO charts), her weight was 24.8 kg (72nd centile, 0.58 SDS according to WHO charts), and her BMI was 18.43 kg/m^2^ (93rd centile, 1.48 SDS according to WHO charts). The parents did not report any signs of fatigue or physical disabilities. She had an appropriate dentition for age, and premature loss of deciduous teeth was not reported. The girl underwent radiologic assessment of the limbs, which demonstrated tibial and fibular deformities, subchondral lucencies in the distal femoral metaphysis, and metaphyseal irregularities (**Figure 5**).

Given the marked radiological findings and the abnormalities in the blodd tests, enzyme replacement therapy with asfotase alfa was initiated at a dose of 2 mg/kg three times per week (total 6 mg/kg/week) not only in the proband, but also in her sister. Asfotase alfa treatment rapidly increased alkaline phosphatase activity to supraphysiologic values and normalized vitamin B6 concentration. After six months, radiological studies demonstrated improved skeletal features in both patients. No adverse events occurred.

**Figure 6:**
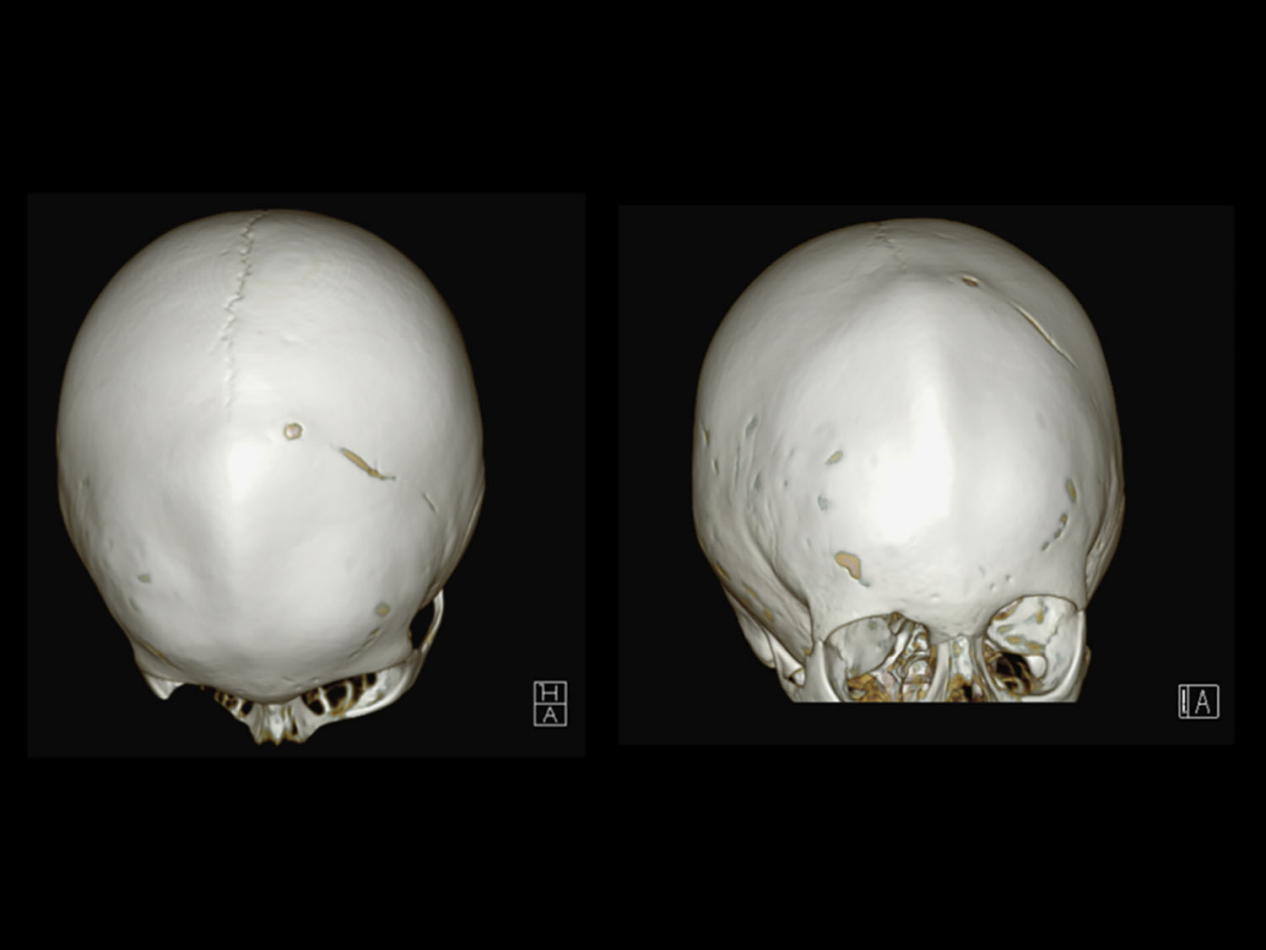
Computed tomography 3D images of the skull of the Family 1 proband, showing the craniosynostosis due to the precocious closure of the metopic and coronal sutures.

Despite continued enzyme replacement therapy, at the age of four, the proband’s cranial morphology progressively deteriorated without cognitive or visual impairment. Neuroimaging revealed craniosynostosis with fusion of the coronal and metopic sutures (**Figure 6**), necessitating ongoing neurosurgical, neurological, and ophthalmological follow-up.

### 2.2 Family 2

**Figure 7:**
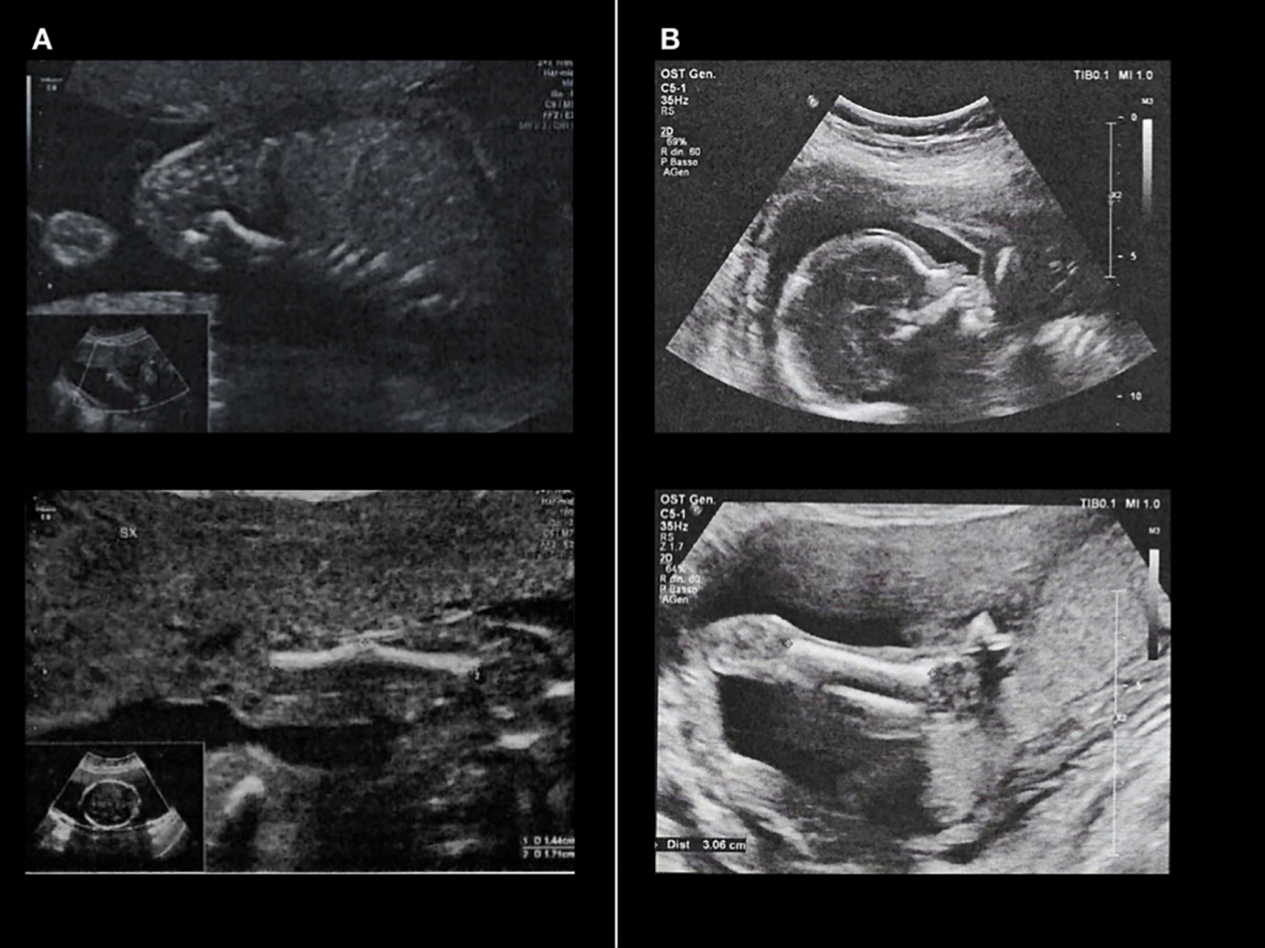
Fetal ultrasound of the two siblings of Family 2. **(A)** shows the images of the first fetus, showing multiple bone deformities and impaired bone mineralization. **(B)** displays images from the second pregnancy, showing a normal-appearing fetus with no skeletal deformities despite identical genotype.

**Figure 8:**
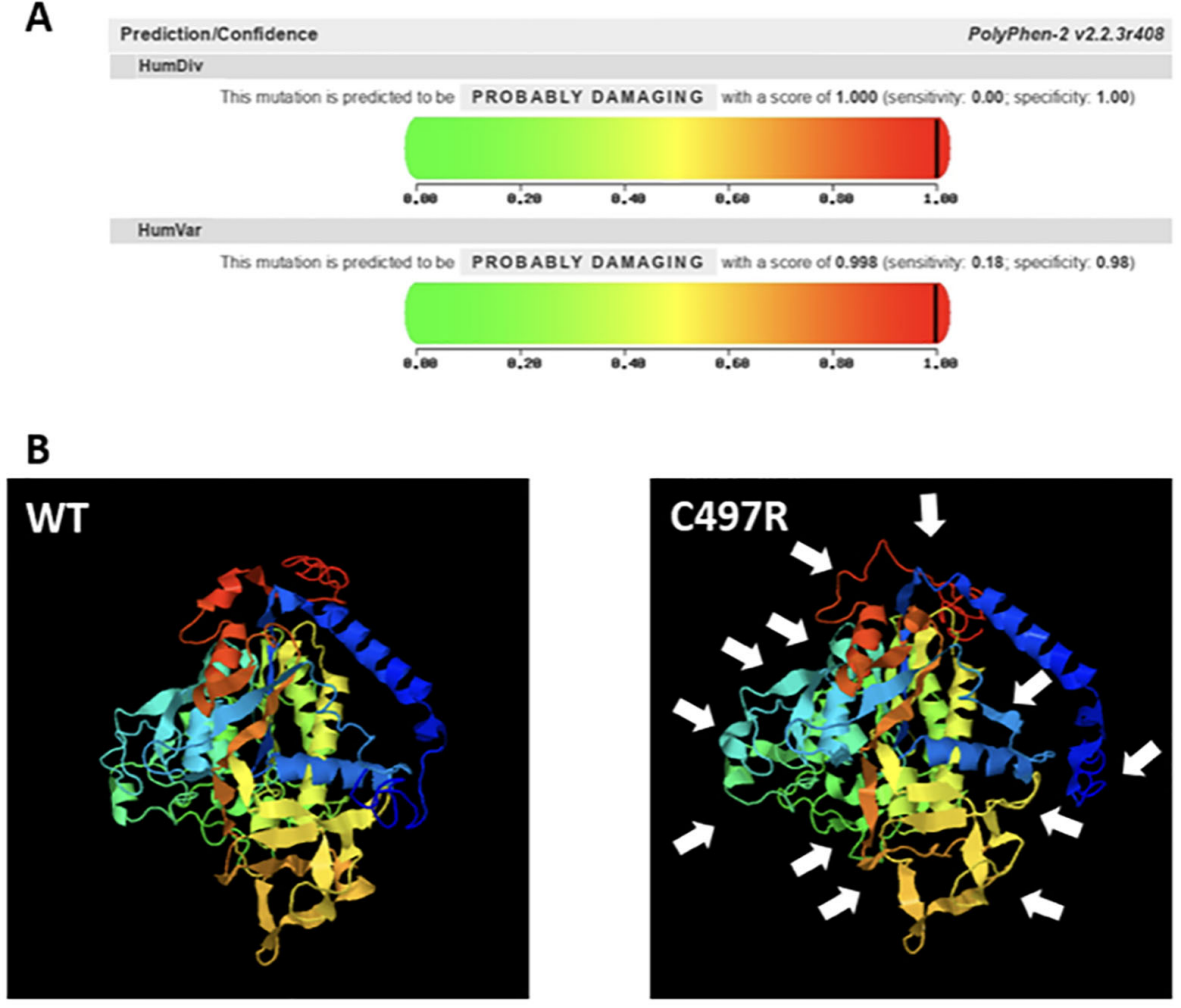
Bioinformatic analysis of the novel missense variant c.1489T>C found in heterozygosity in the Family 2 proband. **(A)** shows the PolyPhen-2 report: the impact of the variant on the function of human TNSALP is very likely damaging. **(B)** depicts the prediction of WT and mutant protein structures obtained by I-TASSER. White arrows show the changes in the structure of the α-helix and β-sheet.

In the first pregnancy of a non-consanguineous couple, the second-trimester ultrasound revealed bilateral humeral bowing of 30 degrees, consistent with potential fractures, and an abnormal chest conformation (**Figure 7**, panel A). Based on the ultrasound findings, osteogenesis imperfecta was suspected. Considering the presence of major malformations, the couple opted for a therapeutic abortion in accordance with the Italian law 194/78. Total body radiography and histological examination of the fetus confirmed the findings of reparative outcomes of multiple fractures and skeletal mineral deficit. Exome sequencing analysis was then performed on fetal tissues, finding two variants of the *ALPL* gene [c.407G>A, p.(Arg136His) classified as pathogenetic, and c.1489T>C, p.(Cys497Arg)]. **Table 2** shows the ACMG classification of the variants found in our two families. The p.(Cys497Arg) is a novel missense variant, which was predicted to be probably damaging according to PolyPhen-2 software (http://genetics.bwh.harvard.edu/pph2/) (**Figure 8**, panel A) and deleterious according to Mutation Taster analysis (http://mutationtaster.org/) (data not shown). We employed I-TASSER (https://zhanggroup.org/I-TASSER/) to predict the three-dimensional structure of the ALP protein. The mutant version displayed numerous structural changes in the α-helix and β-sheet (as shown by the white arrows in **Figure 8**, panel B). The missense mutation at position 497 results in the loss of one of the two disulfide bonds in the ALPL protein, which may account for the phenotype observed in our patients. These cysteine residues are crucial for proper enzyme folding, and disulfide bonds play a significant role in ensuring the structural integrity of TNSALP (16).

The molecular analysis was then extended to both parents. The variant c.407G>A (p.Arg136His) in exon 5 of the *ALPL* gene was identified in the mother, while the variant c.1489T>C (Cys497Arg) in exon 12 of the *ALPL* gene was of paternal origin. In this family, such genotypes correspond to asymptomatic carrier status for the disease; indeed, they showed no clinical signs or symptoms that could be ascribed to it. Both parents had heights above the 50th percentile for the Italian population and had no history of fractures or dental problems. Their biochemical profiles were within normal limits.

A subsequent pregnancy was monitored with prenatal ultrasound examinations, which showed normal fetal development (**Figure 7**, panel B). However, direct sequencing of the relevant *ALPL* exons performed on chorionic villus sampling material confirmed the presence of the same compound heterozygous variants identified in the previous pregnancy (**Figure 3**).

The pregnancy progressed uneventfully, and the infant was delivered via elective cesarean section at 37 + 4 weeks of gestational age. At birth, the infant appeared clinically normal with appropriate birth weight (3.235 kg, 81st centile, 0.86 SDS according to INeS charts), length (48 cm, 49th centile, -0.02 SDS according to INeS charts), and head circumference (33 cm, 43°rd centile, -0.18 SDS according to INeS charts).

Laboratory investigations revealed markedly low serum alkaline phosphatase activity (25 UI/L; normal reference for age and sex > 140 UI/L). Serum calcium, phosphate, parathyroid hormone, and 25OH-vitamin D concentrations were within normal ranges. Serum vitamin B6 concentration was elevated (229 ng/mL; normal range 12.6-45.2 ng/mL).

**Figure 9:**
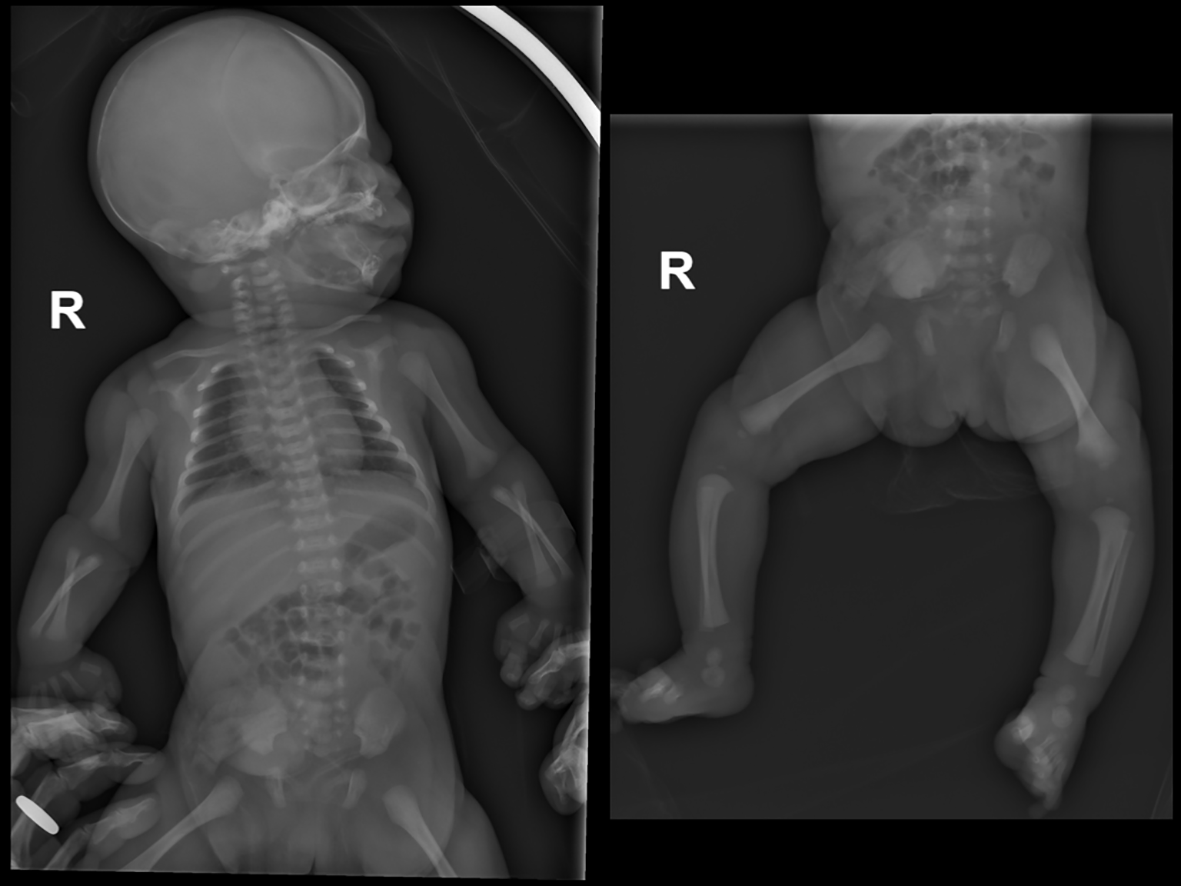
Whole body radiographs of the proband from Family 2 at birth revealed slightly reduced mineralization throughout the entire skeleton.

Total body radiography demonstrated generalized bone hypomineralization with early metaphyseal irregularities (**Figure 9**).

Enzyme replacement therapy with asfotase alfa was initiated at a dose of 2 mg/kg three times weekly, starting on the third day of life. Treatment led to a rapid increase in alkaline phosphatase activity and a prompt normalization of vitamin B6 concentrations. The treatment was well-tolerated, with no cutaneous or systemic adverse events reported.

**Figure 10:**
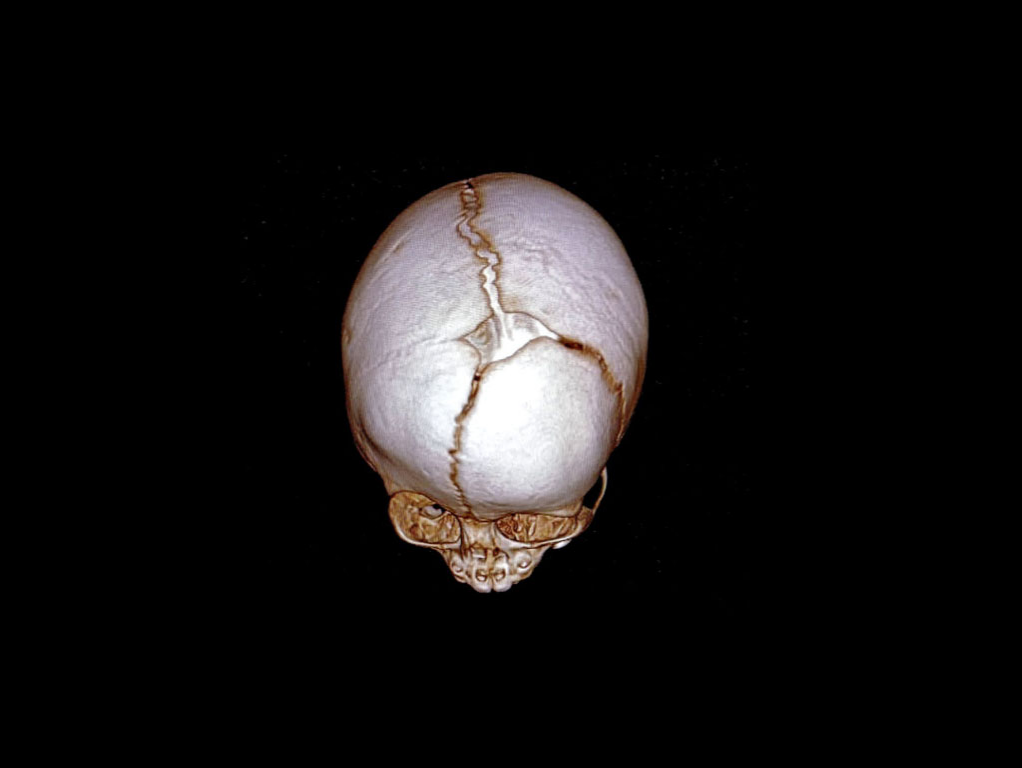
Computed tomography 3D images of the skull of the proband from Family 2, showing the asymmetric craniosynostosis due to the precocious closure of the right coronal suture.

At the age of three months, the patient developed a progressive cranial deformity characterized by marked left-sided frontal bossing and apparent hypoplasia of the left eyebrow arch. Neuromotor development remained within normal limits. At six months of age, a cranial computed tomography (CT) scan revealed right coronal synostosis (**Figure 10**). Decompressive cranioplasty was performed at nine months of age with resolution of craniosynostosis. She is now continuing treatment with asfotase alfa at a dose of 2 mg/kg three times weekly.

## 3 Discussion

Hypophosphatasia is a genetic disorder caused by pathogenic mutations in the *ALPL* gene, leading to decreased activity of the tissue-nonspecific alkaline phosphatase enzyme. The phenotypic spectrum of the disease is very heterogeneous, ranging from extremely severe forms, often lethal in the prenatal or perinatal periods, to very mild forms diagnosed in adult patients (1, 2). Several reports indicated that the affected portion of the enzyme determines the disease phenotype (3, 17–19). In particular, severe alleles were associated with missense mutations localized in the active site, its vicinity, and the homodimer interface (3). The most severe phenotypes are typically transmitted as autosomal recessive traits, while either dominant or recessive traits are responsible for the milder forms (7, 9). Moreover, the number of variants is not a determinant of disease severity (20).

Phenotypic diversity is a common feature of many human diseases (21). Regarding hypophosphatasia, the possibility of different clinical manifestations within the same familial unit leads to an additional diagnostic difficulty in a disease that is still poorly understood. Moreover, the age of onset of symptoms can vary widely, further complicating diagnosis. An additional factor to consider is that different *ALPL* mutations may co-occur, leading to compound heterozygosity. This condition can generate highly variable phenotypes, even within the same families, depending on the specific allelic combination. The factors contributing to this phenotypic diversity among patients with the same *ALPL* mutation remain unknown; each individual likely has a unique interaction between genotype and environmental factors, leading to different clinical outcomes even in close relatives.

The clinical and molecular characteristics of our two families emphasize the phenotypic diversity associated with a single pathogenic variant, as well as the potential for different clinical manifestations among individuals with the same genotype within a single family. We found two pathogenic variants in Family 1, which have been previously reported. The c.571G>A variant was first described in a lethal infantile form of HPP, but it has also been associated with the clinically mild childhood and adult form of the disease, and it can be inherited in an autosomal recessive form in both severe and mild disease (22). The same mutation was reported in a 51-year-old woman who had a history of several metatarsal fractures (23). Finally, a pediatric patient with HPP was a compound heterozygote for c.203C>T and c.571G>A (24). The serum TNSALP activity of the proband’s father, who carried the heterozygous c.571G>A variant, was within the normal range. The authors showed that the ALP activity in 293T cells transfected with *ALPL* c.571G>A was 52.83% of wildtype (24). Interestingly, the mother of Family 1 in our report, who carried the same variant, also showed serum alkaline phosphatase activity within the reference range. This finding highlights that apparently normal ALP values may still reflect reduced enzymatic activity at the molecular level, supporting the concept that standard biochemical measurements may underestimate subclinical functional impairment in heterozygous carriers. This may contribute to the phenotypic variability observed within the same family. The second variant found in Family 1 (c.1258G>A) has been previously described in a patient with perinatal HPP who was homozygote for the mutation and in a patient with childhood HPP who was a compound heterozygote (25). The authors demonstrated that substituting glycine at position 420 decreased the enzyme activity. Specifically, they discovered that the TNSALP variant could not form a functional homodimer, suggesting that glycine 420 may play a direct role in monomer-monomer interactions or be essential for each subunit to attain the correct structure necessary for homodimer formation (25).

Our two familial cases of HPP illustrate a sporadically documented phenomenon characterized by significant phenotypic heterogeneity among relatives with the same genotype. In Family 1, the younger patient exhibits a more severe phenotype compared to her sister, with clear clinical signs and an early onset of symptoms. Conversely, the older sister was diagnosed only after biochemical and molecular analyses were conducted for the entire family. Similar phenotype patterns have been previously described (12, 14, 26, 27). In just two families (12, 14), phenotypic heterogeneity was observed among siblings. In one of these families (12), the heterozygous parents had four children, three of whom were affected by HPP. The second and fourth children were compound heterozygous and received a diagnosis of HPP by the age of two. The older daughter presented with a history of premature loss of deciduous teeth and tooth enamel defects, while the younger sibling exhibited severe failure to thrive, dysphagia, and recurrent vomiting. In a second report (14), the older child of consanguineous parents presented at 4 months of age with severe growth impairment. At 10 months of age, rachitic deformities and significant delays in neuromotor development were apparent. She also exhibited diffuse osteopenia, especially pronounced in the skull. Additionally, she experienced premature loss of her deciduous teeth. Conversely, her younger sister, also affected by HPP, despite sharing the same genotype, showed a milder phenotype, characterized by short stature due to impaired growth velocity.

The diagnosis of HPP in our second family was established on the aborted fetus, which displayed multiple skeletal deformities. The second sibling, who shares the same genotype, experienced normal intrauterine development, and her skeleton showed no signs of hypomineralization or malformation. However, a mild deficiency in bone mineralization was noted at birth, leading to the prompt initiation of enzymatic treatment. Our case is similar to another previously described (13), where the first pregnancy was terminated due to severe fetal hypoplasia and generalized mineralization defects. In contrast, the fetus from the second pregnancy exhibited normal intrauterine growth; however, some signs of skeletal malformation were observed at 34 weeks of gestation. Similarly, enzyme treatment was started shortly after birth in this case as well.

The processes underlying phenotypic variability among family members with the same genotype are still unclear. It may be the effect of other genes, known as modifier genes. These genes can belong to the same gene family, have redundant functions, operate within the same functional pathway, or participate in the formation of protein complexes (28–32). Nonetheless, phenotypic diversity is observed even among isogenic strains of model organisms and in individuals who are genetically “identical,” such as monozygotic twins or cloned cats (33, 34). Additionally, phenotypic variation can occur within genetically identical populations of single cells cultured under the same conditions (35, 36). These instances suggest that stochastic processes may contribute to phenotypic diversity. Stochasticity arises from fluctuations driven by intrinsic, non-deterministic factors in gene expression or molecular interactions, particularly when a very small number of molecules are involved (37).

Our case history highlights that, despite significant advancements in genetic diagnosis, predicting the severity of the phenotype remains challenging. Prenatal diagnosis, in particular, should be interpreted cautiously and must be accompanied by a thorough ultrasonographic examination. Nevertheless, due to the extreme phenotypic heterogeneity, even within families, it is essential to extend the evaluation beyond prenatal findings by integrating genetic investigations with anamnestic, biochemical, and instrumental data. Postnatal assessment, including genetic confirmation and detailed laboratory evaluations, is therefore crucial for more accurately defining the clinical course and guiding appropriate therapeutic decisions.
